# Influence of surface ocean density on planktonic foraminifera calcification

**DOI:** 10.1038/s41598-018-36935-7

**Published:** 2019-01-24

**Authors:** Stergios D. Zarkogiannis, Assimina Antonarakou, Aradhna Tripati, George Kontakiotis, P. Graham Mortyn, Hara Drinia, Mervyn Greaves

**Affiliations:** 1 National & Kapodistrian University of Athens, School of Earth Sciences, Faculty of Geology & Geoenvironment, Department of Historical Geology - Paleontology, Athens, Greece; 2 Department of Earth, Planetary, and Space Sciences, Department of Atmospheric and Oceanic Sciences, Institute of the Environment and Sustainability, California Nanosystems Institute, University of California, Los Angeles, CA USA; 3 European Institute of Marine Sciences (IUEM) Université de Brest, UMR 6538, Domaines Océaniques, Rue Dumont D’Urville, and IFREMER, Plouzané, France; 4 Institute of Environmental Science and Technology, Universitat Autònoma de Barcelona, Barcelona, Spain; 5 Department of Geography, Universitat Autònoma de Barcelona, Barcelona, Spain; 6 Department of Earth Sciences, University of Cambridge, Cambridge, UK

## Abstract

This study provides evidence that ambient seawater density influences calcification and may account for the observed planktonic foraminifera shell mass increase during glacial times. Volumes of weighed fossil *Globigerina bulloides* shells were accurately determined using X-ray Computer Tomography and were combined with water density reconstructions from Mg/Ca and *δ*^18^O measurements to estimate the buoyancy force exerted on each shell. After assessment of dissolution effects, the resulting relationship between shell mass and buoyancy suggests that heavier shells would need to be precipitated in glacial climates in order for these organisms to remain at their optimum living depth, and counterbalance the increased buoyant force of a denser, glacial ocean. Furthermore, the reanalysis of bibliographic data allowed the determination of a relationship between *G. bulloides* shell mass and ocean density, which introduces implications of a negative feedback mechanism for the uptake of atmospheric CO_2_ by the oceans.

## Introduction

Planktonic foraminifera adjust to the dynamic behaviour of the fluid in which they are immersed^1^. As with other zooplankton, the precipitation of relatively heavy calcitic tests, with a specific gravity significantly greater than that of the ambient seawater, provides foraminifera a mechanism to counteract uplifting due to lighter cellular components and allows them to inhabit certain depths, which represent favourable ecological niches, by regulating their buoyancy at the expense of biochemical energy^2^. But little is known about how this behaviour may have varied through time as the density of seawater has evolved in response to changes in the temperature and salinity structure of the oceans. Therefore we explore the novel hypothesis that downcore shell mass variations may in part reflect a hydrostatic response to seawater density changes.

A number of authors have observed that planktonic foraminifera shell mass was higher during glacial stages and lower during interglacials^3–7^. The cause of this behaviour is debated. Since it was first shown in culture experiments that an increase in shell mass was induced by an increase in carbonate ion concentration^8^, a link between calcification efficiency and carbonate ion concentration ([CO_3_^=^]) has been found in the physical environment^9^. A number of authors have shown that glacial shell mass of some planktonic foraminifera species from different regions, is correlated with proxy records for carbonate ion concentration [CO_3_^=^], which increases in seawater due to the decrease of glacial atmospheric *p*CO_2_^3,4,7,10^. This reasoning thus has been used to reconstruct past distributions of carbonate ion concentration from shell mass^5,11,12^.

Additional work has suggested that downcore variations in shell weight may not always be explained by [CO_3_^=^]. For example, a causal relation between shell mass variations and [CO_3_^=^] could not be verified for a number of down-core^6,13,14^ and contemporary biogenic material^15–18^ investigations from different localities. Some authors have suggested that such attempts should be considered with caution^6^. Calcification temperature has been suggested to not always play a major role on shell mass^10^.

Even for modern shells of the same species, in different sites almost identical in water [CO_3_^=^] and temperature, an offset in shell mass has been observed^19^. Although the influence of nutrients has been hypothesized^20^, yet another study^15^ excluded nutrient availability and instead called upon optimum growth conditions as the cause for the observed shell mass variations, although subsequently size-normalized weight was found not to respond to optimum growth conditions^17^. Finally it has been suggested that changes in size-normalized shell weight of planktonic foraminifera reflect mainly abiotic forcing since heavier shells in the Mediterranean were associated with high salinity waters^21^. Dissolution as the major parameter affecting recorded shell mass variation was excluded qualitatively in most of the studies above, while it was semi-quantitatively assessed only for a set of core-top samples^22^.

Since planktonic foraminifera are major contributors of calcium carbonate (CaCO_3_) to the sea floor^23^, the identification of the processes that affect their biomineralization is relevant to testing hypotheses on the origin of glacial-interglacial *p*CO_2_ variations^24^ and to predicting the future of marine calcifying organisms and ecosystems^15^. Here, the state of shell preservation is evaluated by semi-quantitative means, and the role of ocean density variations on shell mass and the subsequent change in the intensity of ocean buoyancy force during glacial-interglacial intervals are examined.

At North Atlantic ODP Site 982 we measured Mg/Ca to reconstruct temperature. Furthermore we measured the volumes of weighed *G. bulloides* shells at different intervals and calculated the buoyancy force exerted to them by the ocean. Reconstructed temperature values were combined with available oxygen isotope data to calculate seawater salinities. The proxy derived salinity and temperature estimates were used in the equation of state of seawater to derive past surface ocean density changes. We also evaluate shell weight and density data from published geochemical datasets for a number of Atlantic and Southwest Pacific sites.

## Results

**Figure 1:**
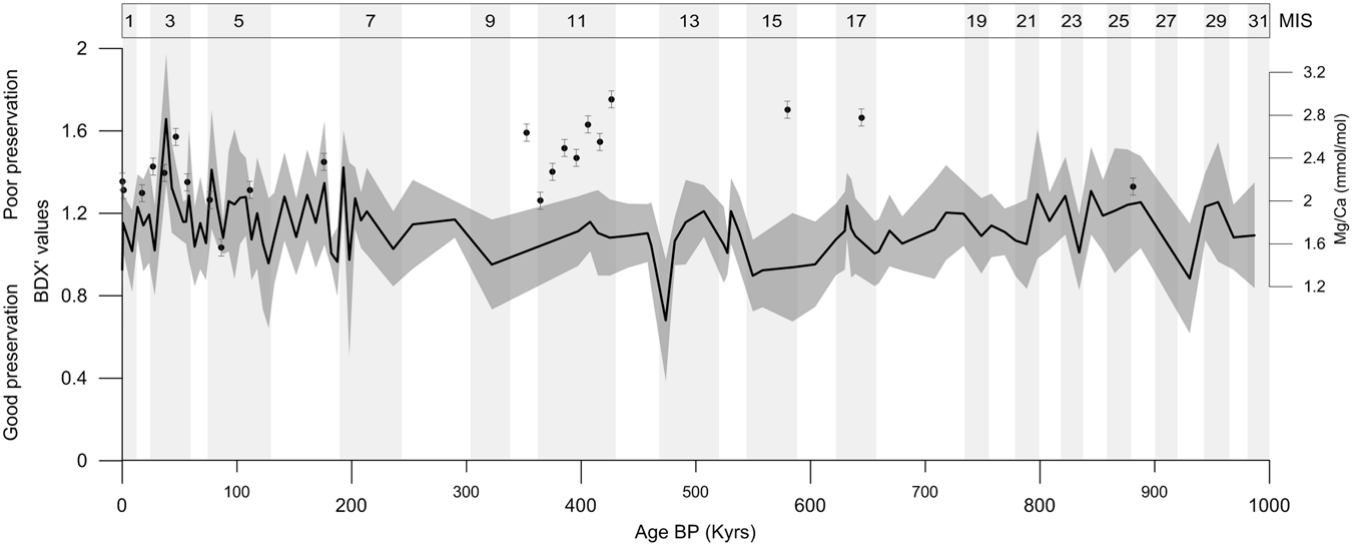
1 million years of a shell preservation index for *Globigerina bulloides* from ODP Site 982 compared to Mg/Ca ratios. Bulloides Dissolution indeX (BDX’) is solid line and circles represent Mg/Ca measurements. Increased BDX’ values signify greater dissolution, with significant dissolution starting at values above 3. The results show no evidence for high dissolution, and also no systematic decrease in Mg/Ca values is observed with reduced preservation. Therefore any low-temperature bias to paleotemperature estimates from Mg/Ca due to preferential post-depositional dissolution of the Mg-rich portions of the foraminiferal calcite is likely to be minimal. The 1*σ* confidence interval for each plot is shown as solid grey area and bars, respectively.

Tests of *G. bulloides* from ODP Site 982 are very well preserved and exhibit evidence for only a minor increase in dissolution during glacial times (Fig. 1), in accordance with previous studies on dissolution impacts on planktic foraminifera from the Atlantic^25^. Preservation events are recorded during stadials. No significant dissolution was inferred for the Mid-Brunhes interval (∼200–600 ka). In particular the assessment of the X-ray imprint of a number of specimens (Table 1) suggests that calcite loss for most may be well below 15%.

**Table 1:** Data summary of specimens used for buoyancy calculations.

ODP 982 MCD	MIS	Age BP (ka)	Individual shell volume (μm^3^)	Number of shells	Shell mass (μg)	Buoyancy •10^−9^ (N)	XDX index
**1.13**	**MIS 3b**	**35.769**	**20,737,310 ± 14%**	**16**	**18.9**	**209.3 ± 15%**	**0.9 ± 0.4**
**1.37**	**MIS 3b**	**48.173**	**21,322,473 ± 13%**	**16**	**17.7**	**215.2 ± 14%**	**0.5 ± 0.3**
**1.57**	**MIS 4**	**58.096**	**20,564,595 ± 12%**	**15**	**18.5**	**207.6 ± 13%**	**0.8 ± 0.3**
**1.73**	**MIS 4b**	**66.035**	**20,395,211 ± 11%**	**16**	**19.0**	**205.7 ± 12%**	**0.8 ± 0.2**
2.07	MIS 5b	82.904	20,059,312 ± 7%	16	17.2	201.6 ± 8%	0.8 ± 0.2
5.63	MIS 8e	287.63	19,961,907 ± 14%	16	15.9	184.7 ± 15%	0.9 ± 0.4
**5.68**	**MIS 8d**	**289.95**	**18,295,153 ± 15%**	**16**	**17.0**	**187.2 ± 15%**	**0.8 ± 0.3**
5.87	MIS 8e	298.77	18,540,906 ± 11%	16	14.4	193.7 ± 12%	0.7 ± 0.3
6.02	MIS 9	304.09	19,190,259 ± 14%	16	15.1	190.4 ± 15%	0.8 ± 0.2
6.07	MIS 9	305.86	18,866,448 ± 10%	16	14.6	180.5 ± 10%	0.8 ± 0.3
6.17	MIS 9	309.41	17,895,821 ± 7%	15	16.4	190.9 ± 8%	0.6 ± 0.3
6.23	MIS 9	311.54	18,903,846 ± 13%	16	16.9	189.0 ± 14%	0.5 ± 0.3

Cold climatic times (glacials, stadials) are shown in bold. MCD = Meters Composite Depth of ODP Site 982 samples, MIS = Marine Isotope Stage and XDX = X-ray Dissolution Index^30^.

**Figure 2:**
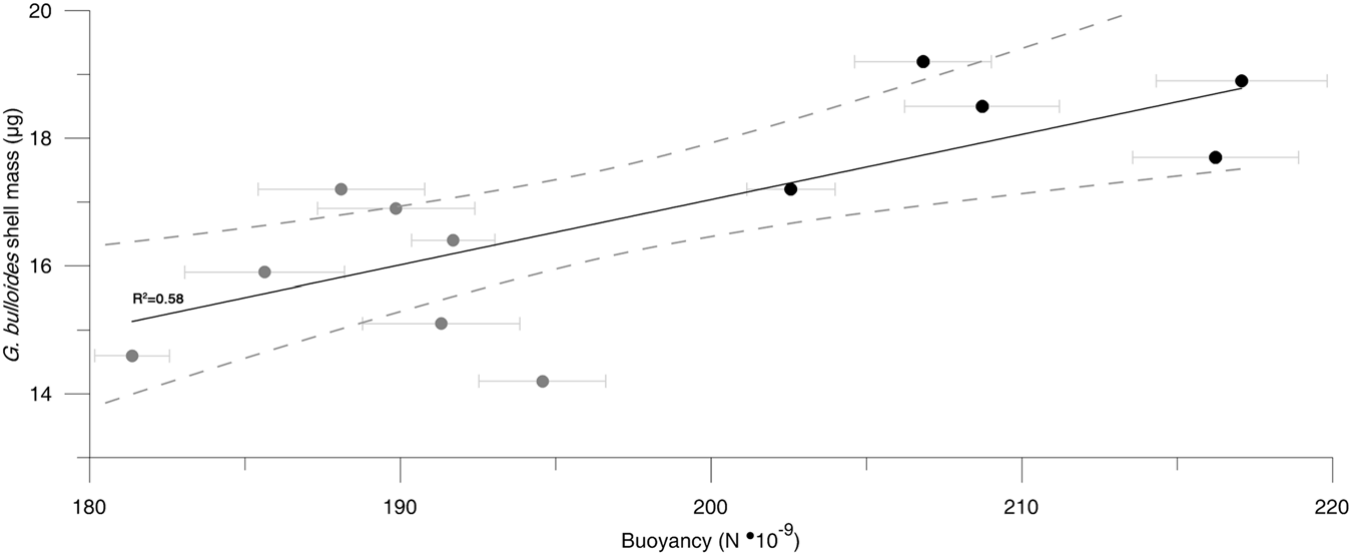
Evidence for a significant correlation between the shell mass of *G. bulloides* from Site 982 and buoyancy force estimates derived from X-ray microtomographic volumetry and combined Mg/Ca - *δ*^18^O measurements of the same samples. Grey dots are values from interglacial or interstadial samples and black dots represent glacial or stadial samples (Table 1). The dashed lines around the regression line indicate the 95% level of confidence.

The buoyancy force exerted on *G. bulloides* by surrounding seawater was estimated as a function of water density. Average buoyancy values calculated for different time intervals were plotted against the mass of contemporary shells and the results are shown in Fig. 2. Calculations of the weight of seawater displaced by the organism were performed with a constant gravitational acceleration (9.82 m/s^2^). We found that foraminifera shell mass correlates well (R^2^ = 0.58, *p* < 0.005, n = 10) with the buoyant force exerted on them by the surrounding seawater suggesting that their shell mass increases with an increase in the force of buoyancy. The heavier shells are the most voluminous ones (Table 1; R^2^ = 0.43, *p* < 0.05, n = 10).

**Figure 3:**
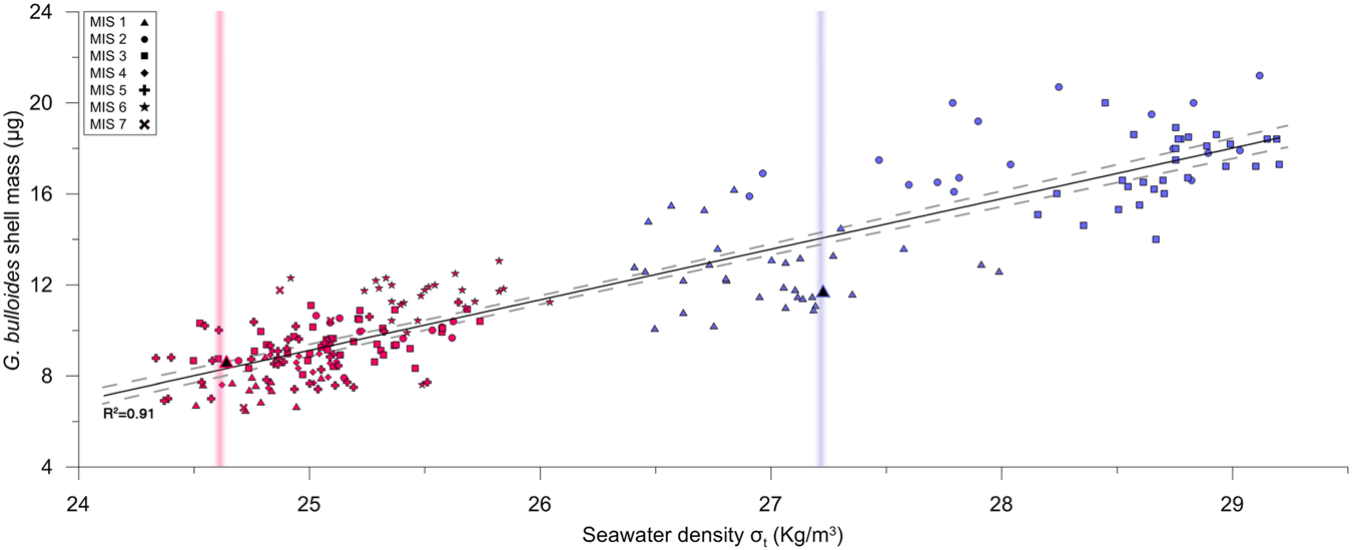
Evidence for a significant correlation between the mass of *G. bulloides* shells from the Eastern Atlantic and seawater paleodensity within two different size classes. The upper right (in blue) data are based on size-normalized weight values for the 300–355 μm size fraction from core NEAP 8K in the Northeast Atlantic. The lower left data (in red) are values from the 250–315 μm sieve fraction for cores MD02‐2594 and MD96‐2080 in the Southeast Atlantic. Ambient seawater paleodensity values are estimated from Mg/Ca and carbonate *δ*^18^O. Core top samples are denoted with bigger, black infilled symbols, while vertical lines represent modern *in-situ* annual average seawater density. The dashed lines around the regression line indicate the 95% level of confidence.

There is evidence of a relationship between *G. bulloides* shell mass and ocean density from the reanalysis of data from core NEAP 8K^4^ which we used to calculate paleodensities. Paleodensity estimates were plotted against *G. bulloides* shell normalized mass, from the 300–355μm size fraction, and the results are shown in Fig. 3. The measurements correlate significantly (R^2^ = 0.56, *p* < 0.001, n = 79) supporting the hypothesis that heavier shells precipitated in denser waters. The correlation between size normalized weight to salinity was R^2^ = 0.52 (*p* < 0.001, n = 79) and to temperature was R^2^ = 0.07 (*p* > 0.1, n = 79), while temperature and salinity in this case were not correlated to each other (*p* > 0.05, n = 79). The significant correlation of shell mass with seawater salinity, in contrast to temperature, supports previous findings^21^. This result implies that the salt concentration of ambient seawater greatly affects plankton shell mass, which could be due to the impact of salinity on density.

Core NEAP 8K covers one glacial-interglacial cycle and the reconstructed seawater density values range from 26.4 to 29.2 Kg/m^3^. These values fall within the range of Atlantic intermediate water density reconstructions from benthic foraminifera across the last deglaciation^26,27^. The data from core NEAP 8K were plotted together with data of different size fractions from South Atlantic MD02‐2594 and MD96‐2080 core records and the results are shown in Fig. 3.

These data show that smaller *G. bulloides* shells, from the 250–315 μm size fraction, record lower seawater densities than shells from a larger size class that are heavier. This can be attributed to calcification at different depths during different ontogenetic life stages of the foraminifera, as has previously been proposed to explain shells of different sizes^28^. *G. bulloides* in its early life stages calcifies in shallower, less dense waters while during later stages of growth by chamber addition sinks into deeper and denser waters, suggesting as well, that larger and hence heavier shells must be precipitated in denser waters. The density values from the South Atlantic cores range from 24.3 to 26.1 Kg/m^3^ and are consistent with previous surface Atlantic water density estimates from planktonic foraminifera across the last deglaciation^29^.

**Figure 4:**
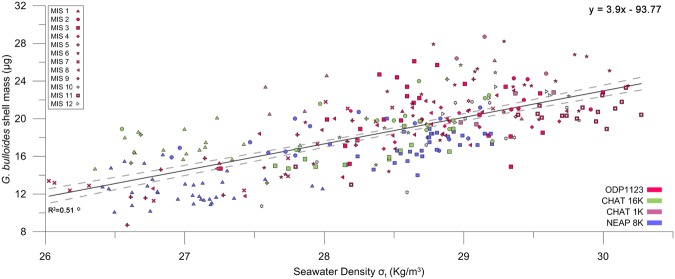
Synthesis of published shell mass data plotted against new estimates of seawater density. Data are for *G. bulloides* shells (300–355 μm) and Mg/Ca - *δ*^18^O derived *in-situ* densities from N. Atlantic and S.W. Pacific sites (*p* < 0.001, n = 312). The dashed lines around the regression line indicate the 95% level of confidence. Data are grouped based on marine isotopic stage (MIS). The correlation between size normalized weight to salinity or temperature alone was R^2^ = 0.44 (*p* < 0.001, n = 315) and R^2^ = 0.03 (*p* > 0.1, n = 315) respectively, while temperature and salinity were uncorrelated (*p* > 0.05).

To further test our hypothesis, we plotted all available published data (see methods) for *G. bulloides* from a restricted size class (Fig. 4). Despite inconsistencies in data acquisition among different data sets, a significant relationship between shell mass and ambient paleo-seawater density (R^2^ = 0.51, *p* < 0.001, n = 312) is verified both at Atlantic and Pacific sites for the past ~435 ky. Thus significant correlation between water density and shell masses of foraminiferal tests is observed for different size fractions, different localities and time spans.

All together, the volumes of individual *G. bulloides* tests were measured and the buoyancy force exerted on these organisms by the ocean, under different climatic regimes, has been calculated. The measurements were performed with the use of a X-ray microcomputed tomography scanner (XMCT). The accuracy of the instrument allows the assessment of shell volume variations under different past oceanographic conditions.

The preservation state of tests was assessed by examining their ultrastructures under the SEM using the Bulloides Dissolution Index (BDX’)^3^, and in some cases internal structures with XMCT using the X-ray Dissolution Index (XDX)^30^, and they were found to be mostly well preserved. Dissolution is not believed to have altered the overall shell volume as any severe dissolution will initially affect the inner calcite layers^31^. In general the mass of the shells is inversely related with their preservation state. The shells were found to be relatively corroded during glacials, when their masses were found to be greater. The fact that the heavier shells were also the most corroded ones, together with the low BDX’ and XDX values, rule out dissolution as the primary controlling factor of the observed shell mass pattern.

For the paleotemperature reconstructions derived from Mg/Ca, the well preserved foraminiferal calcite and the insignificant change of late Pleistocene seawater Mg/Ca concentrations^32^ together with the nearly constant state of calcite saturation state of the ocean since the late Pliocene^33^ significantly rule out processes that have affected skeletal Mg^2+^ record other than biomineralization temperature.

## Discussion

A relationship that equates the biogenically precipitated shell calcite, at different ontogenetic stages, with ambient seawater density was identified in Fig. 3. *G. bulloides* in its early life stages calcifies in shallower, lighter waters and by chamber addition during growth sinks into deeper and denser waters. Because the density and viscosity of sea water are on the order of 10^3^ and 10^2^ times greater than the density and viscosity of air, respectively, foraminifera are much more strongly influenced by buoyant and viscous forces than are comparably sized terrestrial organisms^34^.

The ability of planktonic foraminifera to inhabit specific depths in the surface ocean requires a means for species-specific buoyancy adjustments and calcification control. Test formation is not an inert and solid state but rather a dissolution and reprecipitation process^35^ that allows foraminifera to resorb and reallocate shell calcite during their life cycle^36,37^. The calcite shells provide foraminifera with the negative buoyancy needed to dive to certain depths. The substantial weight of the tests require some mechanism to sustain flotation and permit the foraminifera to adjust their position in the water column. The fibrillar bodies appear to be the most likely cytoplasmic structures mediating buoyancy^38^. In addition to the fibrillar bodies, it is likely that buoyancy is enhanced by the presence of lipid droplets dispersed throughout the cytoplasm^39^.

The behavior of foraminifera to regulate shell mass can also be found in their ability to change test porosity. Porosity affects the density of the test wall since it increases shell buoyancy in waters of low viscosity^40^. Different pore densities (number of pores/surface area) have been found between glacial and interglacial climates^41^. Field and laboratory work conducted on planktonic foraminifera indicate that temperature and salinity affect shell porosity. Warmer and less saline waters both produce lower density seawater where foraminifera construct less dense (more porous) shells as a buoyancy response^42–44^ or for metabolic reasons^45^. Regardless of the actual physiological cause, porosity has be linked to relative seawater densities^46^.

For minor, temporary or short term migrations shallower into the water column, positive buoyancy in plankton may be regulated by alterations in the calcite to protoplasm ratio of the shell^47^ or by the lower-density cytoplasmatic entities that can greatly affect their average living depth^48^. The depth regulating mechanism proposed here may support major migrations over wide density ranges of the water column for the acquisition of a maximum depth during the foraminiferal life cycle, and especially during gametogenesis when additional calcite is secreted. This mechanism is probably present but likely less effective in phytoplankton and other smaller organisms where frictional drag and other surface area dependent forces predominate^2^.

Provided that all other growth controlling factors such as [CO_3_^=^], nutrient availability, temperature, salinity and others are sufficient for optimum growth, planktonic organisms will tend to attain a certain geometry rather than continuously calcifying passively as a function of resource availability. Their shell cannot get unlimitedly thick otherwise its pores would become too long such that gases could no longer diffuse through them. In addition, if tests become too heavy, they might sink in the water column, beyond a critical pressure surface and habitat depth range. The influence of density in depth habitat determination has been manifested elsewhere for most planktonic foraminifera^48^ since it was found that their calcification depths are tuned to particular density layers^49^ or isopycnals^50^.

Optimum habitat depth may be determined by carbonate ion concentration, nutrient availability, hydrography, and/or competition. It is possible to speculate in evolutionary terms, that planktonic calcifiers may have had the advantage of waters with increased [CO_3_^=^] at the times they needed them most. North Atlantic surface waters are, at present, supersaturated with respect to calcite^51^, as most surface ocean waters are^52^. In a glacial ocean, with increased carbonate ion concentration in the surroundings^53^, organisms may need to spend less energy to gather CO_3_^=^ for shell construction and can get heavier more quickly.

The data discussed above suggest that *G. bulloides* shell mass variations during glacial and interglacial times is related to the buoyant force exerted on organisms by surrounding seawater. During glacial times mean ocean density increases due to thermal contraction and storage of fresh water in ice sheets. Sea ice formation affects the salinity of the ocean, while continental ice deposition alters both ocean’s salinity and volume. A denser ocean, with increased salt concentration, will exert greater buoyancy force and thus will tend to buoy plankton towards the sea surface. In order for organisms to counteract this increased buoyant force and maintain hydrostatic equilibrium, they would need to increase shell mass, which in turn would allow them to sink back to their preferred habitat as dictated by hydrography, competition, nutrient availability, and other factors.

Since foraminifera are forced to increase carbonate precipitation during glacial times, then the excess carbonate precipitation alone will decrease the ocean’s total alkalinity or pH and will increase its CO_2_ concentration ([CO_2_])^54^. On the contrary during terminations, when waters are fresher and lower in density, organisms would need to have a lower shell mass than their glacial form in order to maintain their optimal habitat depth within the water column. Such abiotically-driven reduction in planktonic calcification during terminations will decrease the [CO_2_] in seawater, as a by-product of intracellular calcite formation and it may thus provide a mechanism for the ocean to counterbalance increased atmospheric *p*CO_2_.

## Conclusions

The analysis of well-preserved fossil *G. bulloides* shells from ODP Site 982 suggests a relationship between shell mass and ambient seawater density. We propose the hypothesis that this is driven by the need for buoyancy regulation. Changes in the density of waters in the upper water column, as a function of both temperature and salinity may explain some of the observed variation in planktonic foraminifera shell mass during glacial-interglacial transitions. This depth-regulating mechanism adds a new dimension to the debate over what causes downcore shell mass variations. Building on the carbonate ion hypothesis, we speculate these organisms would then have the advantage of heavier shells in glacial waters with higher [CO_3_^=^]. During terminations, abiotically-driven reduction in shell calcification promotes the mechanism of atmospheric CO_2_ uptake by the ocean. Additional work will elucidate whether there are species-specific shell mass-seawater density equations.

## Methods

The present study was conducted on *G. bulloides* specimens from ODP Site 982 that were previously studied^3^ and represent both cold and warm climatic phases. *G. bulloides* was investigated because it was present in all samples from Site 982, and due to the amount of published data already available for the species. Preservation of specimens was assessed via Scanning Electron Microscope (SEM) with the use of a semi-quantitative method, while the total volume of the shells during different time periods was determined with X-ray microcomputed tomography (XMCT) scannings. The determination of foraminifera test volume together with the ambient seawater density, reconstructed from bibliographic data, allowed, according to Archimedes’ principle, the calculation of the buoyancy force of the ocean on the foraminifera shells. The estimation of buoyancy force involved ambient paleo-seawater density calculations, which were based on published *δ*^18^O data and new Mg/Ca measurements. To further test the hypothesis, we examined all available published core datasets that include *G. bulloides* shell Mg/Ca, *δ*^18^O and mass measurements from different Atlantic and Pacific regions. Data were combined to reconstruct ambient seawater densities, and reanalysed to produce graphs of seawater density versus shell mass (Fig. 4). Two-tailed regression analyses were performed using the Reduced Major Axis model, at n-1 degrees of freedom. *n* represents sample size. Standard Bonferroni corrections to significance levels were applied to correct for multiple comparisons.

### Dissolution assessment

The dissolution degree of the ODP 982 specimens that were used in buoyancy calculations (Table 1), for which XMCT scans exist, was assessed by applying the XDX, an empirical dissolution index that evaluates the appearance of dissolution features in the tomographs^30^. The XMCT appearance of their internal structure resembles that of almost intact specimens implying in most of the cases less than 15% of calcite loss^31^. In the absence of XMCT scans for the rest of the record, the carbonate preservation state of samples from ODP Site 982 for the first 1 My was examined, with higher resolution during the late Quaternary, by applying the Bulloides Dissolution Index (BDX’)^55^. In order for the evaluation to be representative of each time slice and the result to be statistically significant, 20 of the previously weighed *G. bulloides* specimens from each downcore sample were assessed. The spiral side of the ultimate chamber of each test was investigated using a ZEISS DSM 940 A Scanning Electron Microscope at the Department of Geosciences, University of Bremen. The BDX’ evaluates the corrosion of shell surface ultrastructure and thus provides a semi-quantitative measure of specimen dissolution^55,56^. These methods have the advantage of directly assessing the dissolution of the specimens rather than their general fragmentation, which is also affected by fragmentation through mechanical sieving or selective dissolution of the finer fragments. The results from the samples where overlapping in the dissolution assessment methods took place suggest that both methods are in good agreement.

### Determination of past seawater density

The combination of Mg/Ca ratios of foraminiferal shell calcite with *δ*^18^O isotope measurements has been used here to reconstruct ambient water salinities^57–59^. Mg/Ca thermometry for core ODP Site 982 was calculated using Anand *et al*.^60^ equation, which is calibrated for temperatures lower than about 10 °C and thus more reliable for reconstructing glacial N. Atlantic temperatures. The use of the same equation is mentioned for the rest of published cores present here except from the temperate latitude cores MD02‐2594 and MD96‐2080 where temperature reconstructions were based on the Mashiotta *et al*.^61^ equation because it yields more accurate results for core-top samples at warmer temperatures.

*G. bulloides δ*^18^O values were adjusted for a ‘vital effect’ using the published offset of 0.52‰^62^. *δ*^18^O_sw_ was calculated using a published paleotemperature equation^63^ and a VPDB-to-SMOW *δ*^18^O conversion of 0.27‰^64^. Salinity estimates were derived from *δ*^18^O_sw_ values using the modern regional salinity-*δ*^18^O relationships^65^, after correcting for global ocean salinity and ice volume changes^66,67^, assuming that these relationships remain constant throughout time^68^.

#### Error propagation in the conversion of Mg/Ca to temperature

The data used in this study are mainly published data, from different laboratories, analysed after different cleaning procedures and precise replicate errors were not published in most cases. For samples from sites other than ODP Site 982 the replicate error (*σ*_Mg/Ca_ = 0.7 °C) and the error of the calibration curve (*σ*_calib_ = 1.1 °C) gives a 1*σ* uncertainty in the temperature estimate of each sample of ±1.2 °C. NEAP 8K is quoted with a typical paleotemperature estimate error of ±1 °C and for cores ODP 1123, CHAT 1K, CHAT 16K the estimated error is on average ±1.3 ^o^C. For ODP core 982 the replicate error based on two splits of eight samples is *σ*_Mg/Ca_ = 0.08 mmol/mol.

*In-situ* seawater densities for particular depths were calculated using the equation of state of seawater^69^ from the temperature and salinity estimates. The Mg/Ca ratios from the present study were combined with published *δ*^18^O isotope measurements^70^ to calculate densities at ODP Site 982 for different time slices. Seawater densities were calculated from available Mg/Ca and *δ*^18^O measurements on foraminifera tests from the 300–355 μm size fraction for core NEAP 8K in the North Atlantic^4^, ODP 1123, CHAT 1K, CHAT 16K in the Southwest Pacific^71^ and from the 250–315 μm size fraction from combined nearby MD02‐2594 and MD96‐2080 core records in the South Atlantic^72^. Together cores MD02‐2594 and MD96‐2080 yielded data for ∼200 kyrs, while the NEAP 8K core covers the last deglacial cycle^73^. For the S. Atlantic shells of the 250–315 μm sieve fraction densities were calculated first for a depth of 75 m, to avoid forcing of the regression with *a priori* lower densities when shallower water depths were used in the equation of state of seawater. Subsequently the regression was recalculated for a more realistic living depth of 25 m^74^ for the smaller foraminifera, which gave a slightly better correlation and is presented in Fig. 3.

#### Propagation of errors in the calculation of in-situ density

The error in the *δ*^18^O of seawater (*σ*_*δw*_) is a combination of the error in the measurement of *δ*^18^O of *G. bulloides* (*σ*_*δc*_ = 0.081‰) and the error in the Mg/Ca-derived pelagic temperature (*σ*_*T*_ = 0.192‰): ${\sigma }_{\delta w}=\sqrt{{\sigma }_{T}^{2}+{\sigma }_{\delta c}^{2}}$, consequently *σ*_*δw*_ = 0.208‰. The total error in the ice-volume–corrected *δ*^18^O of seawater is ${\sigma }_{\delta {\rm{w}} \mbox{-} {\rm{i}}{\rm{c}}{\rm{e}}}=\sqrt{{\sigma }_{\delta w}^{2}+{\sigma }_{SL}^{2}}=0.231\textperthousand$. By assuming a linear relationship between *σ*_δw-ice_ and salinity^65^, the partial differential equations of ref. ^27^ yields an error for salinity *σ*_*S*_ = 0.63 psu and for *in-situ* density *σ*_*t*_ = 1.73 Kg/m^3^.

### Determination of shell volume and buoyancy force

**Figure 5:**
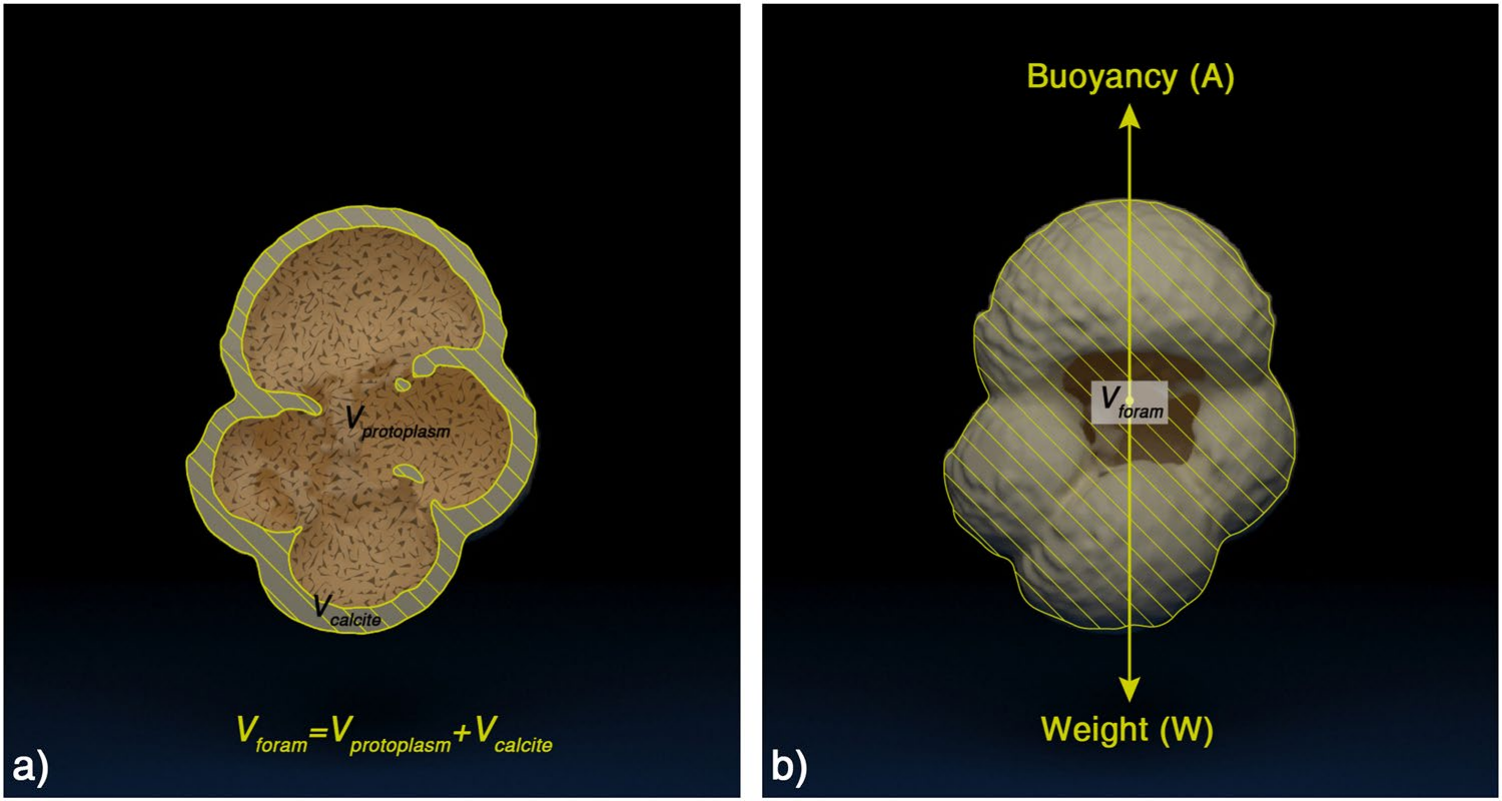
3D rendering of a *G. bulloides* test from the computed tomographic data illustrating (**a**) *V*_*foram*_ and (**b**) the equal action in seawater of the opposing forces of its weight (W) and the ocean’s buoyancy force (A) in order for hydrostatic equilibrium to be attained and for the organism to attain neutral buoyancy at a certain depth.

In order to determine the foraminifera shell volume, X-ray computed tomography (CT) was used. The scannings were performed with a Skyscan 1072 desktop XMCT scanner at the Department of Earth Sciences, University of Cambridge. The scanner uses a point X‐ray source to create a series of radiographs of a sample as it rotates. Cross‐sectional slices (“tomographs”) were reconstructed using Skyscan’s own software that uses the Feldkamp cone‐beam algorithm^75^. The reconstructed tomographs were subsequently processed with CT data visualization software for entire shell volume determination (*V*_*foram*_). In each tomograph the interior of the foraminifera imprint, together with the calcite imprint itself were segmented as a single object. By summing up all cross sections the data visualization software was able to calculate the overall volume (i.e. calcite and cavities) of each shell (Fig. 5).

The available CT data set consisted of scans for foraminifera from 12 different down-core time intervals representing (approximately equal) glacial and interglacial stages of the last three climatic cycles. All samples were scanned under the same conditions: anode voltage was set at 100 kV and the X-ray tube current at 0.98 μΑ. By processing 1024 images per sample a voxel size of ∼1.8 μm was achieved. In total 191 shells were reconstructed from the 12 available down-core time interval samples. On average 16 tests from each sample were scanned and a mean *G. bulloides* shell volume *V*_*foram*_ was calculated for each time interval (Table 1). The total volume *V*_*foram*_ approximates the volume that a living foraminifer would occupy in the water column or the volume, *V*_*dis.water*_, of seawater that it displaces. The buoyant force exerted on the shells was calculated from equation (1) using the estimated water density (*ρ*) and the results are shown in Table 1. The buoyant force (in Newton, N) exerted on a foraminifer (*A*) in equilibrium during floating at its optimum depth equals the weight of the water it displaces (*W*_*displaced*_). The weight of the water that a single foraminifer (without spines) displaces equals it mass (*m*_*dis.water*_) times the acceleration of gravity g (m/s), or is the product of its volume times water density times g. There is an underestimation of the shell volume, both because fossil spines usually break and also because their exact effect on buoyancy is not well understood, but the geochemical signal of the core shell will always reflect ambient seawater properties. Given that *V*_*foram*_ = *V*_*dis.water*_, from above, we get:

1 $$A={W}_{displaced}={m}_{dis.water}\ast g=\rho \ast {V}_{dis.water}\ast g=\rho \ast {V}_{foram}\ast g$$

#### Error propagation in the determination of shell volume and buoyancy force

The error in the calculation of the buoyancy force of the ocean (*σ*_*A*_) is a combination of the error in volume estimate of *G. bulloides* shells (σ_*Vforam*_ = 13.64%) and the error of combined Mg/Ca - δ^18^O derived *in-situ* density (*σ*_*t*_ = 6.27%): ${\sigma }_{A}=\sqrt{{\sigma }_{{Vforam}}^{2}+{\sigma }_{t}^{2}}$, consequently σ_*A*_ = 15%, which is almost equal to the size interval of the sieve fraction used.

## Supplementary information

Dataset 1

Dataset 2
